# Disrupted structural network of inferomedial temporal regions in relapsing–remitting multiple sclerosis compared with neuromyelitis optica spectrum disorder

**DOI:** 10.1038/s41598-022-09065-4

**Published:** 2022-03-25

**Authors:** Eun Bin Cho, Daegyeom Kim, ByeongChang Jeong, Jong Hwa Shin, Yeon Hak Chung, Sung Tae Kim, Byoung Joon Kim, Cheol E. Han, Ju-Hong Min

**Affiliations:** 1 Department of Neurology, College of Medicine, Gyeongsang Institute of Health Science, Gyeongsang National University, Jinju, South Korea; 2 Department of Neurology, Gyeongsang National University Changwon Hospital, Changwon, South Korea; 3 Department of Electronics and Information Engineering, Korea University, Sejong, South Korea; 4 Interdisciplinary Graduate Program for Artificial Intelligence Smart Convergence Technology, Korea University, Sejong, South Korea; 5 Department of Neurology, Samsung Medical Center, Sungkyunkwan University School of Medicine, Seoul, South Korea; 6 Department of Neurology, Neuroscience Center, Samsung Medical Center, Seoul, South Korea; 7 Department of Radiology, Samsung Medical Center, Sungkyunkwan University School of Medicine, Seoul, South Korea; 8 Department of Health Sciences and Technology, Samsung Advanced Institute for Health Sciences & Technology (SAIHST), Sungkyunkwan University, Seoul, South Korea

**Keywords:** Neuroscience, Diseases of the nervous system, Multiple sclerosis

## Abstract

Multiple sclerosis (MS) and neuromyelitis optica spectrum disorder (NMOSD) are two representative chronic inflammatory demyelinating disorders of the central nervous system. We aimed to determine and compare the alterations of white matter (WM) connectivity between MS, NMOSD, and healthy controls (HC). This study included 68 patients with relapsing–remitting MS, 50 with NMOSD, and 26 HC. A network-based statistics method was used to assess disrupted patterns in WM networks. Topological characteristics of the three groups were compared and their associations with clinical parameters were examined. WM network analysis indicated that the MS and NMOSD groups had lower total strength, clustering coefficient, global efficiency, and local efficiency and had longer characteristic path length than HC, but there were no differences between the MS and NMOSD groups. At the nodal level, the MS group had more brain regions with altered network topologies than did the NMOSD group when compared with the HC group. Network alterations were correlated with Expanded Disability Status Scale score and disease duration in both MS and NMOSD groups. Two distinct subnetworks that characterized the disease groups were also identified. When compared with NMOSD, the most discriminative connectivity changes in MS were located between the thalamus, hippocampus, parahippocampal gyrus, amygdala, fusiform gyrus, and inferior and superior temporal gyri. In conclusion, MS patients had greater network dysfunction compared to NMOSD and altered short connections within the thalamus and inferomedial temporal regions were relatively spared in NMOSD compared with MS.

## Introduction

Multiple sclerosis (MS) and neuromyelitis optica spectrum disorder (NMOSD) are chronic inflammatory demyelinating diseases of the central nervous system (CNS) that affect primarily on the brain, spinal cord, and optic nerves. In both diseases, clinical inflammatory relapses are usually accompanied by white matter (WM) lesions, which contribute to brain damage. However, the immunopathogenesis and location of brain tissue damage differ somewhat between MS and NMOSD. Pathological studies have indicated that in contrast to MS, brain lesions in NMOSD are characterized by astrocytopathy with a loss of aquaporin-4 (AQP4) and the absence of cortical gray matter (GM) demyelination, which suggests a different degree of tissue destruction^1,2^. While lesions can occur throughout the brain, MS has distinct magnetic resonance imaging (MRI) features that help distinguish it from other CNS demyelinating diseases, which include a lesion adjacent to the lateral ventricle body or in the inferior temporal lobe, Dawson’s fingers, or a subcortical U-fiber lesion^3^. In contrast, nonspecific small WM lesions are the most common feature in NMOSD, although MS-like lesions reportedly exist in 10–12.5% of cases^4^. However, occult tissue damage also appears in deep GM as well as normal-appearing WM and GM in MS and NMOSD, which is revealed through diffusion tensor imaging (DTI) and volume analyses^1,5,6^.

DTI allows the quantitative evaluation of WM microstructural integrity. Further, graph-theoretical approaches could be applied to the DTI-based connectivity matrix to explore the topological properties of the entire network. Changes in network metrics have been previously revealed in several psychological and neurological disorders such as MS and NMOSD^7–10^. Independent studies indicated decreases in global and local efficiencies in MS^11^ and in the total strength in NMOSD in comparison with healthy controls (HC)^10^. Disrupted WM networks also contributed to impaired cognition in MS and NMOSD patients^8,10^. However, few studies have compared the structural network changes in MS and NMOSD^11,12^. Indirect comparison was obtained, and which showed a greater number of disrupted brain regions in MS compared to NMOSD^12^. While patients with MS had less WM connections compared to HC, those with NMOSD only showed loss of small-word properties compared to HC^11,12^. They suggested that further studies are needed in the same cohort of MS and NMOSD using the same MRI scanner and analytical methods to investigate brain network differences^12^.

Structural connectivity of the brain underlies functional connectivity^13^. In many brain disorders, the presence of predilection sites for brain damage is associated with characteristic changes in structural connectome and symptoms of brain dysfunction^7,9,14^. Therefore, the comparison of MS and NMOSD brains at the connectome level may help to understand pathophysiologic differences beyond visible lesion distribution. In this study, we investigated the organizational differences of the whole-brain networks between MS, NMOSD, and HC groups. We also identified connectome-level differences in the structural networks of the three groups using network-based statistics (NBS), and their associations with clinical variables.

## Results

Table 1 summarizes the demographics and clinical features of RRMS (*N* = 68), NMOSD (*N* = 50), and 26 HC. The NMOSD group was older than the MS and HC groups (*p* < 0.001 and *p* = 0.004, respectively), but there were no differences in sex. The disabilities of patients were also more severe in the NMOSD group than in the MS group (*p* < 0.001). Brain attacks were more prevalent in MS than in NMOSD (cerebral hemisphere: 100% vs 44%, *p* < 0.001; brainstem/cerebellum: 65% vs. 46%, *p* = 0.043). Neither patient group had gadolinium enhancement in their cerebral lesions. Each MS patient had brain lesions in more than one MS-typical locations, such as adjacent to lateral ventricles body, in the inferior temporal lobes or subcortical U-fibers. Among patients with NMOSD, 56% (*N* = 28) of patients had more than one NMOSD-typical brain lesions. The imaging findings of NMOSD patients in our study were summarized in Supplementary Table S1. Supplementary Figure S1 shows the brain lesion probability maps for the MS and NMOSD groups. MS lesions were more often immediately adjacent to the lateral ventricles, and more prevalent than NMOSD lesions, especially in the corpus callosum and the body, posterior horn, and inferior horn of the lateral ventricles.

**Table 1:** Demographics and clinical characteristics of study subjects.

	MS (n = 68)	NMOSD (n = 50)	HC (n = 26)	*p* value
Sex, *N* of females (%)	51 (75.0)	42 (84.0)	21 (80.8)	0.481^a^
Age, years, mean ± SD	34.7 ± 8.7	44.4 ± 11.6	35.3 ± 11.3	< 0.001^b^
Disease duration, years, median (IQR)	3.9 (1.725–7.275)	2.75 (1.175–12.075)	NA	0.467
AQP4-IgG positivity, *N* (%)	0 (0)	45 (90.0)	NA	< 0.001
Attack numbers, median (IQR)	2 (1–4)	3 (2–5)	NA	0.129
**Anytime involvement**^**c**^**, *N* (%)**			NA	
Cerebral hemisphere	68 (100)	22 (44.0)		< 0.001
Brainstem/cerebellum	44 (64.7)	23 (46.0)		0.043
Spinal cord	52 (76.5)	38 (76.0)		0.953
Optic nerve	27 (39.7)	28 (56.0)		0.080
EDSS, median (IQR)	1 (0–2.0)	2.5 (1.5–6.0)	NA	< 0.001
Use of drugs^d^, *N* (%)	57 (83.8)^e^	45 (90.0)^f^	NA	0.333

*MS* multiple sclerosis, *NMOSD* neuromyelitis optica spectrum disorder, *HC* healthy control, *EDSS* expanded disability status scale, *IQR* interquartile range, *NA* not applicable.

^a^*p* value from chi-squared test.

^b^*p* value from ANOVA, posthoc tests results: MS vs. NMOSD (*p* < 0.001), HC vs. NMOSD (*p* = 0.004), and MS vs. HC (*p* > 0.05).

^c^Symptomatic involvement.

^d^Taken at the time of brain MRI.

^e^Interferon β-1b (n = 24), interferon β-1a (n = 11), teriflunomide (n = 8), azathioprine (n = 5), fingolimod (n = 3), glatiramer acetate (n = 3), dimethyl fumarate (n = 2), mitoxantrone (n = 1).

^f^Azathioprine (n = 29), mycophenolate mofetil (n = 12), rituximab (n = 2), cyclosporine (n = 1), methotrexate (n = 1).

### Disrupted brain network topology in MS and NMOSD

Overall, the MS and NMOSD groups had lower total strength, clustering coefficient, global efficiency, and local efficiency and had longer characteristic path length (CPL) than HC, but there were no differences between the MS and NMOSD groups (Table 2). Disease duration or Expanded Disability Status Scale (EDSS) score in the MS and NMOSD groups had positive correlation with CPL and negative correlation with the other global properties of network (Table 3). Since the longer CPL is coincident with the lower global efficiency, all showed the same trend. When compared with the HC group, brain regions with altered nodal characteristics were more widespread in the MS group than in the NMOSD group. The left medial orbital region of superior frontal gyrus had lower nodal degree and nodal strength and the left superior parietal gyrus, and precuneus had lower nodal strength in the MS and NMOSD groups compared with the HC group. The left fusiform gyrus and the right superior occipital gyrus in MS also had lower nodal strength compared with HC. Furthermore, significant differences were indicated among the nodal measures of several brain regions between the MS and NMOSD groups. The local efficiencies of the left hippocampus, left parahippocampal gyrus, left superior temporal gyrus, and right Heschl’s gyrus, and the regional efficiency of the left middle frontal gyrus were significantly lower in MS than in NMOSD. The brain regions with significant between-group differences within nodal measures are listed in Supplementary Table S2. The correlation results between nodal measures of the brain regions and clinical parameters were listed in Supplementary Table S3.

**Table 2:** Comparison of global network topological measures between MS, NMOSD and HC groups.

Network measures	MS	NMOSD	HC	Three group comparison^a^	MS < HC^b,c^	NMOSD < HC^b,c^	MS < NMOSD^b,c^
Total strength	299.197 ± 33.021	298.906 ± 40.273	324.588 ± 26.187	0.005	< 0.001	0.012	0.931
Clustering coefficient	0.187 ± 0.016	0.191 ± 0.016	0.202 ± 0.013	< 0.001	< 0.001	0.041	0.132
Characteristic path length	4.750 ± 0.462	4.769 ± 0.695	4.383 ± 0.254	0.005	< 0.001	0.013	0.672
Local efficiency	0.340 ± 0.028	0.343 ± 0.028	0.370 ± 0.020	< 0.001	< 0.001	0.002	0.506
Global efficiency	0.237 ± 0.020	0.238 ± 0.023	0.256 ± 0.014	< 0.001	< 0.001	0.003	0.953

*MS* multiple sclerosis, *NMOSD* neuromyelitis optica spectrum disorder, *HC* healthy control; Data area expressed as mean ± standard deviation.

^a^*p* values from the permutation-based ANCOVA controlling for age and sex between three groups (MS, NMOSD, HC).

^b^A < B, decreased values in A relative to B.

^c^FDR adjusted *p* values of post-hoc tests based on the permutation based ANCOVA controlling for age and sex.

**Table 3:** The associations between global network measures and clinical parameters (disease duration and EDSS) in the MS and NMOSD groups.

Network topological measures	MS				NMOSD			
	Disease duration		EDSS		Disease duration		EDSS	
	Beta	*p* value	Beta	*p* value	Beta	*p* value	Beta	*p* value
Total strength	− 2.3647	0.0185	− 3.6249	0.1360	− 1.9158	0.0463	− 6.4236	0.0013
Clustering coefficient	− 0.0011	0.0143	− 0.0020	0.0736	− 0.0009	0.0102	− 0.0030	< 0.0001
Characteristic path length	0.0309	0.0243	0.0800	0.0144	0.0301	0.0719	0.1111	0.0013
Local efficiency	− 0.0020	0.0159	− 0.0044	0.0280	− 0.0016	0.0143	− 0.0051	0.0002
Global efficiency	− 0.0014	0.0192	− 0.0029	0.0463	− 0.0013	0.0234	− 0.0038	0.0010

*MS* multiple sclerosis, *NMOSD* neuromyelitis optica spectrum disorders, *EDSS* expanded disability status scale.

### Disrupted subnetworks in MS and NMOSD

**Figure 1:**
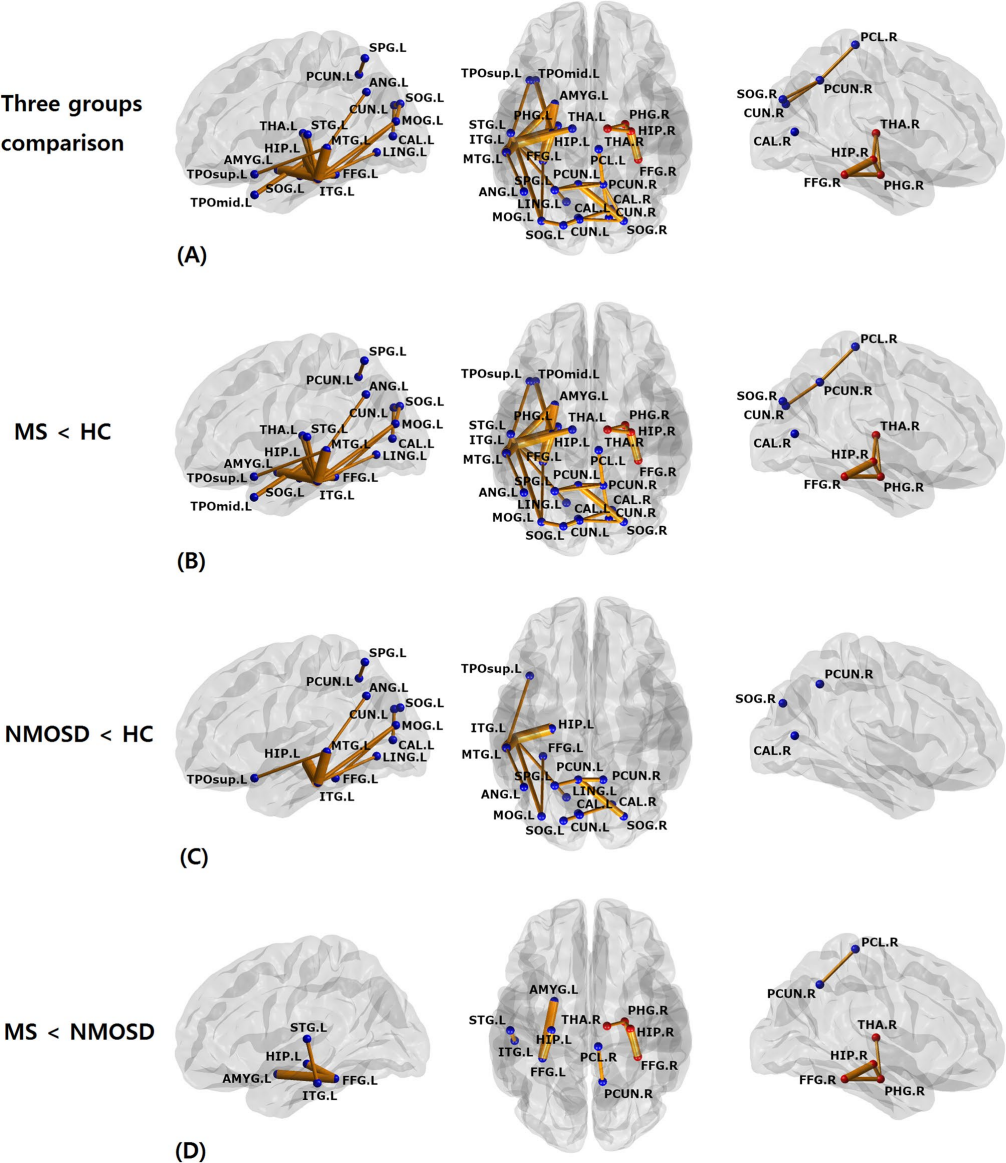
The two disrupted subnetworks in the multiple sclerosis (MS) and neuromyelitis optica spectrum disorder (NMOSD) groups as identified through network-based statistics. Significantly different connections between (**A**) three groups (MS, NMOSD, healthy controls [HC]), (**B**) MS and HC, (**C**) NMOSD and HC, and (**D**) MS and NMOSD were displayed; each edge was disrupted in MS or NMOSD compared with HC and there were no edges with increased edge weights in NMOSD compared with MS. The left column displays the lateral view of the left hemisphere, the middle column displays the transverse views of both hemispheres, and the right column displays the lateral view of the right hemisphere. The blue and red circles are the brain regions of subnetworks 1 and 2, respectively. The orange lines are the edges that connect each region. *HIP* hippocampus, *PHG* parahippocampal gyrus, *AMYG* amygdala, *CAL* calcarine fissure, *CUN* cuneus, *LING* lingual gyrus, *SOG* superior occipital gyrus, *MOG* middle occipital gyrus, *FFG* fusiform gyrus, *SPG* superior parietal gyrus, *ANG* angular gyrus, *PCUN* precuneus, *PCL* paracentral lobule, *THA* thalamus, *STG* superior temporal gyrus, *TPOsup* temporal pole: superior temporal gyrus, *MTG* middle temporal gyrus, *TPOmid* temporal pole: middle temporal gyrus, *ITG* inferior temporal gyrus.

NBS identified two disrupted subnetworks (threshold = 3.0) among the MS, NMOSD, and HC groups (Table 4, Fig. 1A, Supplementary Fig. S2). The first subnetwork (subnetwork 1, *p* < 0.001) consisted of regions mostly located in the left temporo-parieto-occipital lobes, although the precuneus, cuneus, superior occipital gyrus, and calcarine cortex had bilateral involvement. Post-hoc tests indicated that compared with HC, the MS group had significant decreases in most edges of subnetwork 1 (28 out of 29 edges); however, in NMOSD only about half of the edges (13 out of 29 edges) were significantly decreased (Fig. 1B,C). Some edges were more disrupted in the MS group than in the NMOSD group, which were the connections between the left hippocampus and fusiform gyrus, between the fusiform gyrus and amygdala, between the superior temporal and inferior temporal gyri, and between the right precuneus and paracentral lobule (Fig. 1D). The second subnetwork (subnetwork 2, *p* = 0.028) was only disrupted in the MS group and was generally located in the inferomedial temporal region and consisted of five connections between the hippocampus, parahippocampal gyrus, fusiform gyrus, and thalamus in the right hemisphere. Compared with the NMOSD group, most edges (four out of five edges) were significantly disrupted in the MS group. We also identified that several edges of the disrupted subnetworks were associated with disease duration or EDSS score in the MS and NMOSD groups (Supplementary Table S4).

**Table 4:** The post-hoc test result for the identified subnetworks through network-based statistics.

Subnetwork	Edges	MS < HC^a,b^	NMOSD < HC^a,b^	MS < NMOSD^a,b^
1	Hippocampus_L-ParaHippocampal_L	0.0120	0.3675	0.1476
	Hippocampus_L-Fusiform_L	0.0016	0.4264	0.0016
	Hippocampus_L-Temporal_Pole_Mid_L	0.0003	0.0809	0.6019
	Hippocampus_L-Temporal_Inf_L	0.0003	0.0027	0.1291
	ParaHippocampal_L-Fusiform_L	0.0117	0.4330	0.0648
	Amygdala_L-Fusiform_L	0.0006	0.3211	0.0023
	Amygdala_L-Temporal_Inf_L	0.0006	0.1159	0.0960
	Fusiform_L-Temporal_Inf_L	0.0009	0.0944	0.0944
	Lingual_L-Temporal_Inf_L	0.0015	0.0156	0.5481
	Angular_L-Temporal_Mid_L	0.0042	0.0156	0.8146
	Thalamus_L-Temporal_Inf_L	0.0009	0.0943	0.1657
	Temporal_Sup_L-Temporal_Inf_L	0.0108	0.5087	0.0330
	Temporal_Mid_L-Temporal_Inf_L	0.0003	0.0036	0.3916
	Angular_L-Temporal_Mid_L	0.0042	0.0156	0.8146
	Temporal_Pole_Sup_L-Temporal_Mid_L	0.0132	0.0149	0.8908
	Parietal_Sup_L-Precuneus_L	0.0027	0.0248	0.8553
	Parietal_Sup_L-Precuneus_R	0.0060	0.1915	0.1915
	Precuneus_L-Precuneus_R	0.0045	0.0075	0.6966
	Precuneus_R-Paracentral_Lobule_R	0.0144	0.2193	0.0335
	Cuneus_R-Precuneus_R	0.0060	0.1610	0.3292
	Occipital_Sup_R-Precuneus_L	0.0015	0.0024	0.1714
	Occipital_Sup_R-Precuneus_R	0.0629	0.4413	0.0629
	Calcarine_L-Cuneus_L	0.0138	0.0360	0.6150
	Calcarine_R-Occipital_Sup_L	0.0003	0.0362	0.5915
	Cuneus_L-Occipital_Sup_L	0.0015	0.2552	0.1632
	Cuneus_L-Occipital_Sup_R	0.0183	0.0928	0.1210
	Occipital_Sup_L-Occipital_Mid_L	0.0030	0.1429	0.3463
	Occipital_Mid_L-Fusiform_L	0.0057	0.0121	0.9828
	Occipital_Mid_L-Temporal_Inf_L	0.0045	0.0060	0.7591
2	Hippocampus_R-ParaHippocampal_R	0.0189	0.9795	0.0189
	Hippocampus_R-Fusiform_R	0.0033	0.9492	0.0006
	Hippocampus_R-Thalamus_R	0.0021	0.0546	0.5244
	ParaHippocampal_R-Fusiform_R	0.0120	0.7685	0.0120
	ParaHippocampal_R-Thalamus_R	0.0243	0.9313	0.0440

*MS* multiple sclerosis, *NMOSD* neuromyelitis optica spectrum disorders, *HC* healthy controls, *Temporal_Pole_Mid* Temporal pole: middle temporal gyrus, *Temporal_Inf* Inferior temporal gyrus, *Temporal_Mid* Middle temporal gyrus, *Temporal_Sup* Superior temporal gyrus, *Temporal_Pole_Sup* Temporal pole: superior temporal gyrus, *Parietal_Sup* Superior parietal gyrus, *Occipital_Sup* Superior occipital gyrus, *Occipital_Mid* Middle occipital gyrus, *R* right; *L* left.

^a^A < B, decreased edges in A relative to B.

^b^FDR adjusted *p* values of post-hoc tests based on the permutation based ANCOVA controlling for age and sex.

## Discussion

Disrupted global topological organization of WM networks was demonstrated in both MS and NMOSD, with no differences between these two conditions. However, at the nodal level, the MS group had more brain regions with altered metrics than did the NMOSD group when compared with the HC group. Furthermore, the left hippocampus, left parahippocampal gyrus, left superior temporal gyrus, and right Heschl’s gyrus of the MS group had greater decreases in local efficiencies compared with the NMOSD group. Two distinct subnetworks were identified to characterize the disease groups. Disconnected edges were more widespread in the MS group compared with the NMOSD group, and discriminative connectivity changes were mostly found in the thalamus and inferomedial temporal regions. Network alterations were associated with EDSS score and disease duration in both groups.

### Disorganized WM network in MS and NMOSD

The whole-brain WM networks of MS and NMOSD were characterized by reduced total strength, global efficiency, and local efficiency compared with HC. These changes were also correlated with higher EDSS scores and longer disease durations in both disease groups, which is consistent with previous studies^11,15^. Similar patterns of change in structural connectivity have been reported in MS^11,16,17^ and NMOSD^12,18^. Our previous study reported that patients with NMOSD only had reduced total strength compared with HC, which was likely due to including fewer patients with cerebral lesions (14% vs 44% in this study)^10^.

### More disrupted regions with altered network topology in MS than NMOSD

Brain regions with altered nodal topologies were more widely distributed in MS than in NMOSD. Decreased nodal degree or nodal strength with connection losses was indicated for MS in the left fusiform gyrus and several other brain hub regions, such as the left medial orbital part of the superior frontal gyrus, precuneus, and superior parietal gyrus, and the right superior occipital gyrus^19^. Decreased nodal degree or strength was also indicated for NMOSD in the left medial orbital part of the superior frontal gyrus, precuneus, and the right superior occipital gyrus. Many of the connections to and from hub regions were long-range association fibers, which are more susceptible to WM damage and disconnection from brain disorders^14^. Medial orbital part of the superior frontal gyrus receives various sensory input and is associated with reward, mood and emotion; the lower connectivity with parahippocampal gyrus/medial temporal lobe might contribute to reduced happy memories and anhedonia^20^. Precuneus, superior parietal and superior occipital gyri are functional and structural hubs important in global communication and associated with memory, visuospatial function, or object recognition^21^. The fusiform gyrus is the largest component of the ventral temporal cortex and is also a conduit for long association fibers such as the inferior longitudinal fasciculus and inferior frontal occipital fasciculus^22^. Dysfunction and atrophy of the fusiform gyrus occurred in MS^23,24^. Another study using graph theoretical analysis and diffusion MRI found that MS was associated with a larger number of nodes with reduced nodal strength (26 out of 84 nodes) compared with HC, more than what the present study indicated (5 out of 90 nodes)^17^. This may be due to our patients having shorter disease durations with less brain damage, or differences in analysis methods (e.g., construction of WM tractography and the number of comparison groups). Similar to the present study, previous studies have also found that MS was associated with a larger number of disrupted regions than NMOSD^11,12^ and similar brain regions with decreased efficiency were found in MS^11^ and NMOSD^12^ compared with HC. However, these studies did not directly compare structural networks between MS and NMOSD. Therefore, it was particularly interesting that the differences between the MS and NMOSD patients of the present study were primarily in the local efficiencies of several temporal regions; the left hippocampus, left parahippocampal gyrus, left superior temporal gyrus, and right Heschl’s gyrus exhibited significantly smaller local efficiencies in MS than in NMOSD. These regions had a role in memory, visuospatial/auditory processing, language and social cognition^25,26^ and were susceptible to microstructural damage or atrophy in MS even compared with NMOSD^27,28^ Previously, MS patients exhibited severe cognitive impairment, especially verbal and visual memory, compared with NMOSD patients^29^, which might be associated with left superior temporal gyrus volume loss^30^.

### Disrupted subnetworks within the thalamus and inferomedial temporal regions differentiating MS from NMOSD

Two subnetworks that characterized the structural connectivity abnormalities in the MS and NMOSD groups were also identified. Subnetwork 1 consisted of broad regions in the temporo-parieto-occipital lobes (mostly on the left side) and encompassed a part of default-mode, visual/visuospatial, and memory systems. Disrupted edges included decreased strength nodes, which were the left fusiform gyrus, left precuneus, left superior parietal gyrus, and the right superior occipital gyrus. Several disrupted edges, including the connection between the right and left precuneus, were significantly associated with EDSS score or disease duration in both disease groups. Subnetwork 2 consisted of five edges between the hippocampus, parahippocampal gyrus, fusiform gyrus, or thalamus in the right hemisphere, which were the right-side counterparts of those in subnetwork 1. The weights of most edges (four out of five) were negatively associated with the duration of MS. A previous study of WM structural networks in MS found decreased efficiency in brain regions related with default-mode, visual, memory, and language function^11^. Within these subnetworks, all connections except for one were disrupted in the MS group. However, in the NMOSD group there was relatively less disruption of the short connection between the thalamus and inferomedial temporal regions, including edges between the thalamus, hippocampus, parahippocampal gyrus, amygdala, fusiform gyrus, and inferior and superior temporal gyri. These regions overlapped with brain regions that had decreased local efficiencies in MS than in NMOSD.

These findings suggest that the pathological alterations in the corresponding GM regions and their surroundings were greater in MS than NMOSD, which was supported by a previous report that the GM volume of the left parahippocampal, superior temporal gyri, and the right hippocampus was smaller in MS than in NMOSD, while GM atrophy was diffuse in MS compared to HC^28^. In RRMS patients, both cortical and deep GM atrophies were reportedly associated with the disrupted integrity of the connected WM tracts, with the associations being strongest in the temporal lobes such as the hippocampus, parahippocampal gyrus, fusiform gyrus, and lateral temporal regions^31^. The present study supports previous reports that demyelination and atrophy of the thalamus and hippocampus was evident in MS patients^1^, and lesions in the inferior temporal lobe that were common in MS were helpful to be distinguished from NMOSD^3^.

There were several limitations in this study. First, brain network alterations in NMOSD might vary between patients since brain involvement does not occur in all patients. Second, the duration of MS could affect the network comparison results since edge weights between the hippocampus, parahippocampal gyrus, fusiform gyrus, and thalamus—which differentiated MS from NMOSD—were negatively correlated with MS duration. Third, inclusion of patients within 3 months after myelitis relapse (11% and 28% of MS and NMOSD patients, respectively) may affect the correlation between EDSS score and network measures. Fourth, the HC group was not exactly age-matched with the patient groups, especially the NMOSD group, which may confound the comparison results. Fifth, the tractography resulted in a longer *z*-direction, which may have affected its overall quality since the voxel dimensions of our DTI protocol were anisotropic (1.72 mm × 1.72 mm × 3 mm). Finally, deterministic tractography is subject to a major inherent limitation of the crossing-branching problem.

The present study compared connectome-level differences in structural networks between MS, NMOSD, and HC. WM network disruption was more widespread and severe in MS than in NMOSD. Significant disconnections within inferomedial temporal regions might be a differentiated feature in MS from NMOSD.

## Methods

From the prospective cohort of CNS demyelinating disease at Samsung Medical Center in Seoul, South Korea, patients with relapsing–remitting MS (RRMS, *N* = 68) and NMOSD (*N* = 50), who visited the clinic between January 2014 and December 2018, were enrolled by retrospective chart review. Inclusion criteria were patients who (1) over the age of 18 years, (2) met the revised 2017 McDonald criteria for RRMS^32^ or revised 2015 NMOSD diagnostic criteria^33^, and (3) had analyzable brain MRI performed at least 3 months after relapse in the brain. The NMOSD patients included 45 (90%) with AQP4-IgG, which was measured with a cell-based indirect immunofluorescence assay as previously described^34^. AQP-IgG-negative NMOSD patients were also seronegative for myelin oligodendrocyte glycoprotein-IgG, which was determined by in-house live cell-based immunofluorescence assay using an anti-human IgG1-Fc secondary antibody, as previously described^35^. At the time of selection, 8 (11%) MS patients and 14 (28%) NMOSD patients had experienced a spinal cord attack within the previous 3 months, while the rest were all in remission. We also included 26 HC who had no history of medical, neurological, or psychiatric disorders. Of those, 21 people were recruited from a previous study^10^ and the rest were prospectively enrolled. This study was approved by the Institutional Review Board of Samsung Medical Center (IRB No. 2020-04-119), and written informed consent was obtained from all participants. All procedures were performed in accordance with relevant guidelines and regulations.

### Image acquisition

All participants underwent a three-dimensional volumetric brain MRI scan. An Achieva 3.0-Tesla MRI scanner (Philips, Best, the Netherlands) was used to acquire 3D T1 Turbo Field Echo MRI data using a sagittal slice thickness of 1.0 mm, over contiguous slices with 50% overlap and no gap, a repetition time (TR) of 9.9 ms, an echo time (TE) of 4.6 ms, a flip angle of 8° and matrix size of 240 × 240 pixels reconstructed to 480 × 480 over a field of view of 240 mm. 3D fluid attenuated inversion recovery (FLAIR) MRI data were acquired in the axial plane with the following parameters: axial slice thickness of 1 mm, no gap; TR 11,000 ms; TE 125 ms; flip angle 90°; and matrix size of 512 × 512 pixels. In the whole-brain DTI, sets of axial diffusion-weighted single-shot echo-planar images were collected with the following parameters: 128 × 128 acquisition matrix; 1.72 × 1.72 × mm^3^ voxels; 70 axial slices; 220 × 220 mm^2^ field of view; TR 7696 ms, TE 60 ms; flip angle 90°; slice gap 0 mm; b-factor of 600 s/mm^2^. With the baseline image without diffusion weighting (the reference volume), DTI were acquired from 45 different directions. All axial sections were acquired parallel to the anterior commissure–posterior commissure line and perpendicular to the mid-sagittal plane.

### Image preprocessing and network construction

We defined brain regions as network nodes using the automated anatomical labeling atlas^36^, which consists of 78 cortical and 12 subcortical regions, excluding cerebellum regions. We registered them onto the DTI space of each subject using Statistical Parametric Mapping software (version 12, SPM12)^37^. The overall procedure is briefly introduced as follows. We first obtained deformation fields between the DTI space and the tissue probability map (TPM) space through segmentation procedure. In the segmentation procedure, we used average sized TPM template and mutual information-based affine registration. Using the obtained deformation fields, we normalized the DTI of each subject onto the TPM space. After we co-registered the AAL atlas to the TPM space, the AAL atlas, transformed in the TPM space, was projected onto the standard space by applying the inverse of the deformation fields obtained in the segmentation procedure; we matched its voxel size with the original DTI by reslicing it. In the normalization and co-registration procedure of the AAL atlas, we used the nearest neighborhood interpolation method that led clearer boundaries of regions. As a result, we obtained the registered AAL atlas in each subject’s DTI.

We defined network edges by obtaining the averages of fractional anisotropy (FA) values of all voxels on the streamlines connecting any two brain regions. We first performed eddy-current correction of FSL’s Diffusion Toolkit (version 3.0) to adjust unwanted movements by registering all volumes with gradient directions to the reference volume. The gradient directions were appropriately rotated during this alignment procedure^38^. We performed tractography using the Fiber Assignment by Continuous Tracking algorithm with 45 degrees as the angular threshold determined using Diffusion Toolkit with TrackVis^39,40^ on the movement adjusted DTI^38^. We then collected all the voxels on the streamline connection paths and obtained the averages of their FA values. WM voxels where FA values of the seed voxels exceeded 0.2 were included and streamlines shorter than 20 mm were excluded. As a result, we developed a 90-by-90 connectivity matrix consisting of 90 brain regions whose edge weights were the mean FA values between all pairs of brain regions.

### Network topology

We quantified global and nodal properties of network using the Brain Connectivity Toolbox (http://sites.google.com/site/bctnet)^41^. We measured degree, strength, clustering coefficient, characteristic path length (CPL), local efficiency, global efficiency, and regional efficiency.

#### Degree

Degree is the number of neighboring nodes linked to a node. When an edge between the ith and jth node exists, ${a}_{ij}=1$; otherwise, ${a}_{ij}$ = 0. The degree of the ith node, ${K}_{i}$, is computed by summing all values of ${a}_{ij}$ between the ith node and others, where *N* is the number of the nodes in network.

$${K}_{i}= \sum_{j\in N}{a}_{ij}.$$

#### Strength

Strength at the nodal level, nodal strength, is computed by summing all edge weights linked to a node. The nodal strength of the ith node, ${S}_{i}$, is defined by the summation of ${w}_{ij}$, where ${w}_{ij}$ is the edge weight between the ith and jth node. Strength at the global level, total strength, is computed by summing all edge weights in whole network.

$${S}_{i}= \sum_{j\in N}{w}_{ij}.$$

#### Clustering coefficient

Clustering coefficient measures how well nodes are clustered in a network using the number of triangles between those nodes^42^. In a weighted network, the total intensity of triangles is used instead of the number of triangles. The total intensity of triangles of the ith node, ${t}_{i}$, is computed by the summation of the cubic root of products of all edge weights for all connected triangles, where j and k are the indices of its neighboring nodes^43^.

$${t}_{i}= \sum_{j,k\in N}{({w}_{ij}{w}_{jk}{w}_{ki})}^{1/3},$$

where ${w}_{ij}, {w}_{jk}$, and ${w}_{ki}$ indicated edge weights between two neighboring nodes linked to the ith node. The clustering coefficient at the nodal level, nodal clustering coefficient is defined by dividing the total intensity of the ith node by the number of possible triangles linked to ith node, that is, ${K}_{i}({K}_{i}-1)/2$.

$${C}_{i}= \frac{2{t}_{i}}{{K}_{i}({K}_{i}-1)}.$$

The clustering coefficient at the global level is defined by averaging of the nodal clustering coefficients over all nodes.

#### Characteristic path length (CPL)

CPL is defined as the average of shortest path lengths for all pair of nodes in a network^42^. We transformed path length between two nodes as reciprocal of the edge weight connecting them, because the edge weight in our network represents the strength of connection between two nodes. Then, we used the Dijkstra algorithm to find shortest path length between any pair of nodes. If the edge between two nodes is disconnected, because the reciprocal of edge weight is infinite, we ignored them in averaging.

$$L = \frac{\sum_{{d}_{ij}\ne \infty }{d}_{ij}}{D},$$

where ${d}_{ij}$ is the shortest path length between the ith and jth nodes, and D is the number of finite ${d}_{ij}$.

#### Local and global efficiency

Local efficiency is defined as the average efficiency of local subgraphs where the local subgraph is the set of neighboring nodes linked to a certain node excluding the centered node^44^. It captures the efficient communication between neighbors of a certain node. We defined local efficiency of nodal level by averaging them^44^. The local efficiency at the global level is defined as the average of the nodal local efficiency of all the nodes^44^.

$${E}_{loc, nodal}(i)= \frac{1}{2}\sum_{i\in N}\frac{\sum_{j,k\in N, j\ne i, k\ne i}{({w}_{ij}{w}_{ik}{{[d}_{jk}]}^{-1})}^{1/3}}{{K}_{i}({K}_{i}-1)}.$$

Global efficiency is defined by the average of the reciprocal of shortest path lengths between all pairs of nodes^44^.

$${E}_{glob}= \frac{1}{N}{\sum }_{i\in N}\frac{{\sum }_{j\in N, j\ne i}{d}_{ij}^{-1}}{N-1} .$$

#### Regional efficiency

Regional efficiency defined as the average of reciprocal of the shortest path length between a certain node and all the other nodes in a network. It shows how well a node communicates with all the others.

In weighted network, path length between two nodes represents a reciprocal of edge weight between them.

$${E}_{reg}\left(i\right)= \frac{1}{\left(N-1\right)}\sum_{i\ne j\in N}\frac{1}{{d}_{ij}},$$

where the shortest path length between the *i*th and *j*th node, ${d}_{ij}$, is computed using Dijkstra algorithm.

### Edge screening

Since the connectivity matrices obtained through tractography may include false-positive and false-negative connections, their effects were controlled using a method proposed by de Reus and van den Heuvel^45^. We first computed prevalence rate of each connection. The prevalence rate is a ratio of the number of subjects for which a certain connection exists within the HC group. As an example, if all the subjects in the HC group have a certain connection, the prevalence rate of the connection is one. We identified whether a certain connection actually exists using a group threshold which is set with a value between 0 and 1. As an example, if the prevalence rate of a certain connection is bigger than a chosen group threshold, the connection is considered as an existing connection. Otherwise, it is considered as a non-existing connection. After deciding which connection is an existing connection, we defined the false positive and false negative as follows: if a non-existing edge exists in a subject, it is a false positive; if an existing edge does not exist in a subject, it is a false negative. As a chosen group threshold increases, since the number of existing connections increases, the number of false positives decreases, and that of the false negatives increases. We counted the number of false positives and false negatives for various group thresholds and chose a balancing point which minimizes both the number of the false positives and false negatives as the optimal group threshold value, which was 0.66 in our data set. Connections that were weaker than the optimal group threshold were not considered connections.

### Network based statistics

To identify a subnetwork that consisted of significant differences in WM connections between the MS, NMOSD, and HC groups, NBS^46^ was performed with analysis of covariance (ANCOVA) while controlling for the effects of age and sex. Within NBS, cluster-based thresholding of statistical maps was performed to control the familywise error rate during mass univariate testing of every network connection. We thresholded the test statistics from ANCOVA, and the connected edges formed subnetworks (clusters). NBS also used permutation testing to estimate the significance levels of group differences according to the number of larger subnetworks when compared with randomly formed subnetworks. NBS therefore extracted subnetworks with significantly different connections between the three groups. Thresholds were selected when stable clusters form over multiple runs. The threshold was 3.0 and 10,000 permutations were performed.

### Lesion probability map

Lesions from the FLAIR images were automatically segmented using the lesion prediction algorithm^47^ implemented using the Lesion Segmentation Toolbox (LST) (version 3.0.0, www.statistical-modeling.de/lst.html) for SPM12 (https://www.fil.ion.ucl.ac.uk/spm/). This estimated the lesion probability of each voxel using a logistic regression model that was trained using the data of 53 MS patients with severe lesion patterns. We applied this model to each voxel within the FLAIR images to segment the lesions and estimate the lesion probability. Voxels with lesion probabilities below 50% were discarded and binarized to develop a lesion map image for every subject. We then registered the lesion map images to the Montreal Neurological Institute standard-space template. The averages of each patient group were calculated to help develop the group lesion probability map.

### Statistical analyses

Network measures were compared using permutation-based ANCOVA^48,49^ which controlled for the effects of age and sex. Because 90 nodes received group comparisons for nodal measures, multiple comparison corrections were performed using the false discovery rate (FDR)^50,51^.

Since ANCOVA does not determine differences between specific groups, post-hoc tests were performed using three pairwise permutation-based ANCOVA, and then FDR was used to perform multiple comparison corrections on these three pairwise comparisons. Identical post-hoc tests were also performed on the subnetwork edges obtained from NBS. *N* was set as 10,000 for permutation-based ANCOVA and number of permutations.

We also investigated the association between topological network characteristics and clinical variables using the generalized linear model. We controlled for the effects of age and sex:

$$Clinical\, variables= {\beta }_{0} + {\beta }_{1}\times network\, measures+{\beta }_{2}\times age + {\beta }_{3} \times sex,$$

where each beta value is the regression coefficient of each term. Significance levels of the regression coefficient of network measures (${\beta }_{1})$ were determined through ANCOVA.

Statistical analyses were performed with SPSS Version 20.0 (IBM Corp, Armonk, NY, USA), Matlab (version 2019a, MathWorks, Natick, MA, USA) and in-house software programs.

## Supplementary Information

Supplementary Tables and Figure Legends.

Supplementary Figure S1.

Supplementary Figure S2.
