# Supplementary material for: Disrupted structural network of inferomedial temporal regions in relapsing–remitting multiple sclerosis compared with neuromyelitis optica spectrum disorder
Source: Sci Rep. 2022 Mar 25;12:5152. doi: 10.1038/s41598-022-09065-4 (PMC8956623; doi:10.1038/s41598-022-09065-4)
Supplement: Supplementary file 1 — Supplementary Tables and Figure Legends. [file 41598_2022_9065_MOESM1_ESM.docx]

**Table S1** Summary of brain lesions in patients with NMOSD based on 2015 NMOSD diagnostic criteria.

| Imaging findings | Frequency, *N* (%) |
| --- | --- |
| Typical lesions |  |
| Area postrema | 9 (18) |
| Dorsal brainstem/cerebellum | 13 (26) |
| Diencephalon | 4 (8) |
| Adjacent to lateral ventricle/diffuse corpus callosum | 13 (26) |
| Long corticospinal tract | 9 (18) |
| Hemispheric | 6 (12) |
| Non-typical small lesions | 6 (12) |
| No lesion | 16 (32) |

NMOSD, neuromyelitis optica spectrum disorder

**Table S2** Nodal topological differences between MS, NMOSD and HC groups.

| Nodal degree | Nodal strength | Clustering coefficient | | Local efficiency | | Regional efficiency | |
| --- | --- | --- | --- | --- | --- | --- | --- |
| MS, NMOSD < HC^a^ | MS, NMOSD < HC^a^ | MS, NMOSD < HC^a^ | MS < NMOSD^a^ | MS, NMOSD < HC^a^ | MS < NMOSD^a^ | MS, NMOSD < HC^a^ | MS < NMOSD^a^ |
|  |  |  |  | **Temporal_Inf_L**/R |  | **Temporal_Inf_L** |  |
|  | Fusiform_L |  |  | **Fusiform_L**/R |  | **Fusiform_L** |  |
|  |  | Hippocampus_L/R | Hippocampus_R | **Hippocampus_L**/R | Hippocampus_L | Hippocampus_L/R |  |
|  |  | **ParaHippocampal_L** |  | **ParaHippocampal_L**/R | ParaHippocampal_L | **ParaHippocampal_L**/R |  |
|  |  | Amygdala_R |  | Amygdala_L |  | **Amygdala_L** |  |
|  |  | Temporal_Pole_Mid_L/R | Temporal_Pole_Mid_R | Temporal_Pole_Mid_L/R |  | **Temporal_Pole_Mid_L/R** |  |
|  |  | Temporal_Pole_Sup_R |  | **Temporal_Pole_Sup**_L/**R** |  | **Temporal_Pole_Sup_R** |  |
|  |  | Temporal_Sup_L |  | **Temporal_Sup_L**/R | Temporal_Sup_L | Temporal_Sup_R |  |
|  |  | Temporal_Mid_L |  | **Temporal_Mid_L/R** |  | Temporal_Mid_R |  |
|  |  |  |  | Heschl_L/R | Heschl_R | **Heschl_L**/R |  |
|  | **Precuneus_L** |  |  | **Precuneus_L/R** |  | **Precuneus_L/R** |  |
|  |  |  |  | **Angular_L** |  | **Angular_L** |  |
|  |  |  |  | Paracentral_Lobule_L/R |  | Paracentral_Lobule_L/R |  |
| Parietal_Sup_L | **Parietal_Sup_L** |  |  | **Parietal_Sup_L/R** |  | **Parietal_Sup_L/R** |  |
|  |  |  |  | **Parietal_Inf_L/**R* |  | **Parietal_Inf_L** |  |
|  |  |  |  | SupraMarginal_L |  | **SupraMarginal_L/R** |  |
|  |  |  |  | **Postcentral**_L/**R** |  | Postcentral_L |  |
|  | Occipital_Sup_R |  |  | **Occipital_Sup**_L/**R** |  | **Occipital_Sup_L/R** |  |
|  |  |  |  | **Occipital_Mid_L** |  | Occipital_Mid_L |  |
|  |  | Calcarine_L |  | **Calcarine_L** /R |  | **Calcarine_L/R** |  |
|  |  |  |  | **Cuneus_L**/R |  | **Cuneus_L/R** |  |
|  |  |  |  | Lingual_L/R |  | Lingual_L/R |  |
|  |  |  |  | **Cingulum_Ant_L** |  | **Cingulum_Ant_L/R** |  |
|  |  |  |  | **Cingulum_Mid_L** |  | **Cingulum_Mid_L** |  |
|  |  |  |  | **Cingulum_Post_L/R** |  | Cingulum_Post_L |  |
|  |  |  |  | Precentral_L |  | **Precentral_L**/R |  |
|  |  |  |  |  |  | Supp_Motor_Area_L/R |  |
|  |  |  |  | Frontal_Sup_L |  |  |  |
|  |  | **Frontal_Sup_Orb_L**/R | Frontal_Sup_Orb_R | **Frontal_Sup_Orb_L** |  | Frontal_Sup_Orb_R* |  |
|  |  |  |  | Frontal_Sup_Medial_L |  | **Frontal_Sup_Medial_L** |  |
|  |  |  |  | Frontal_Mid_L |  | Frontal_Mid_L/R | Frontal_Mid_L |
|  |  | Frontal_Inf_Tri_L/R | Frontal_Inf_Tri_R | **Frontal_Inf_Tri_L**/R |  | Frontal_Inf_Tri_R* |  |
|  |  |  |  | **Frontal_Inf_Oper_L** |  | Frontal_Inf_Oper_L* |  |
|  |  |  |  | Rolandic_Oper_L |  |  |  |
|  |  |  |  | **Frontal_Mid_Orb_L** |  | Frontal_Mid_Orb_L |  |
|  |  |  |  | **Frontal_Inf_Orb_R** |  |  |  |
| **Frontal_Med_Orb_L** | **Frontal_Med_Orb_L** |  |  |  |  | **Frontal_Med_Orb_L** |  |
|  |  | **Rectus_L** |  | **Rectus_L** |  | Rectus_L*/**R** |  |
|  |  |  |  |  |  | **Olfactory_L** |  |
|  |  |  |  | **Caudate_L** |  | **Caudate_L** |  |
|  |  | Thalamus_L | Thalamus_L |  |  | Thalamus_R |  |

^a^A < B, decreased nodal measures in A relative to B

Unmarked words, only MS < HC; words with asterisk, only NMOSD < HC; boldface words, MS and NMOSD < HC

MS, multiple sclerosis; NMOSD, neuromyelitis optica spectrum disorder; HC, healthy controls; R, right; L, left;

Parietal_Sup, Superior parietal gyrus; Occipital_Sup, Superior occipital gyrus; Frontal_Med_Orb; Superior frontal gyrus, medial orbital; Temporal_Inf, Inferior temporal gyrus; Temporal_Pole_Mid, Temporal pole: middle temporal gyrus; Temporal_Pole_Sup, Temporal pole: superior temporal gyrus; Temporal_Sup, Superior temporal gyrus; Temporal_Mid, Middle temporal gyrus; Parietal_Sup, Superior parietal gyrus; Parietal_Inf, Inferior parietal, but not supramarginal and angular gyri; Occipital_Sup, Superior occipital gyrus; Occipital_Mid, Middle occipital gyrus; Cingulum_Ant, Anterior cingulate and paracingulate gyri; Cingulum_Mid, Median cingulate and paracingulate gyri; Cingulum_Post, Posterior cingulate gyrus; Frontal_Sup, Superior frontal gyrus, dorsolateral; Frontal_Sup_Orb, Superior frontal gyrus, orbital part; Frontal_Sup_Medial, Superior frontal gyrus, medial; Frontal_Mid, Middle frontal gyrus; Frontal_Inf_Tri, Inferior frontal gyrus, triangular part; Frontal_Inf_Oper, Inferior frontal gyrus, opercular part; Rolandic_Oper, Rolandic operculum; Frontal_Mid_Orb, Middle frontal gyrus, orbital part; Supp_Motor_Area, Supplementary motor area

**Table S3** The associations between nodal network measures and clinical parameters (disease duration and EDSS) in the MS and NMOSD group

| Network measures | Nodes | MS | | | | NMOSD | | | |
| --- | --- | --- | --- | --- | --- | --- | --- | --- | --- |
|  |  | Disease duration | | EDSS | | Disease duration | | EDSS | |
|  |  | beta | *p* value | beta | *p* value | beta | *p* value | beta | *p* value |
| Nodal strength | Occipital_Sup_R | -0.1221 | 0.0423 |  |  |  |  | -0.4069 | 0.0002 |
|  | Precuneus_L |  |  |  |  |  |  | -0.4321 | 0.0153 |
| Clustering coefficient | Frontal_Inf_Tri_R | -0.0023 | 0.0194 |  |  |  |  |  |  |
|  | Hippocampus_L |  |  |  |  | -0.0024 | 0.0011 | -0.0043 | 0.0087 |
|  | Hippocampus_R | -0.0022 | 0.0167 |  |  |  |  | -0.0040 | 0.0145 |
|  | Thalamus_L |  |  |  |  |  |  | -0.0039 | 0.0118 |
|  | Temporal_Sup_L |  |  |  |  |  |  |  |  |
|  | Temporal_Pole_Sup_R |  |  | -0.0039 | 0.0338 |  |  | -0.0040 | 0.0045 |
|  | Temporal_Mid_L |  |  |  |  | -0.0014 | 0.0457 |  |  |
|  | Temporal_Pole_Mid_L |  |  |  |  |  |  | -0.0051 | 0.0075 |
|  | Temporal_Pole_Mid_R |  |  |  |  |  |  | -0.0049 | 0.0035 |
| Local efficiency | Precentral_L |  |  |  |  | -0.0017 | 0.0207 |  |  |
|  | Frontal_Sup_L |  |  |  |  | -0.0014 | 0.0225 |  |  |
|  | Frontal_Sup_Orb_L |  |  | -0.0069 | 0.0153 |  |  |  |  |
|  | Frontal_Mid_L |  |  | -0.0048 | 0.0408 | -0.0016 | 0.0139 |  |  |
|  | Frontal_Inf_Oper_L |  |  |  |  | -0.0024 | 0.0076 |  |  |
|  | Frontal_Inf_Tri_R | -0.0021 | 0.0289 | -0.0059 | 0.0089 |  |  | -0.0056 | 0.0041 |
|  | Frontal_Inf_Orb_R |  |  |  |  |  |  | -0.0081 | 0.0002 |
|  | Rolandic_Oper_L | -0.0020 | 0.0468 |  |  |  |  |  |  |
|  | Frontal_Sup_Medial_L |  |  | -0.0102 | 0.0001 | -0.0038 | 0.0002 | -0.0101 | 0.0000 |
|  | Cingulum_Ant_L | -0.0033 | 0.0058 |  |  | -0.0030 | 0.0020 | -0.0063 | 0.0021 |
|  | Cingulum_Mid_L | -0.0046 | 0.0008 | -0.0071 | 0.0351 | -0.0035 | 0.0010 | -0.0074 | 0.0011 |
|  | Cingulum_Post_L | -0.0040 | 0.0033 |  |  | -0.0041 | 0.0008 | -0.0090 | 0.0006 |
|  | Cingulum_Post_R |  |  |  |  | -0.0043 | 0.0005 | -0.0099 | 0.0002 |
|  | Hippocampus_L | -0.0027 | 0.0142 |  |  | -0.0022 | 0.0320 | -0.0064 | 0.0031 |
|  | Hippocampus_R | -0.0032 | 0.0048 | -0.0073 | 0.0063 |  |  | -0.0063 | 0.0005 |
|  | ParaHippocampal_L | -0.0029 | 0.0210 |  |  |  |  | -0.0056 | 0.0122 |
|  | ParaHippocampal_R |  |  |  |  |  |  | -0.0061 | 0.0057 |
|  | Calcarine_L | -0.0038 | 0.0015 |  |  | -0.0034 | 0.0058 | -0.0069 | 0.0094 |
|  | Calcarine_R |  |  |  |  | -0.0031 | 0.0205 | -0.0073 | 0.0098 |
|  | Cuneus_L | -0.0035 | 0.0032 |  |  |  |  | -0.0073 | 0.0047 |
|  | Cuneus_R | -0.0028 | 0.0160 |  |  | -0.0030 | 0.0143 | -0.0096 | 0.0001 |
|  | Lingual_L | -0.0027 | 0.0199 |  |  | -0.0032 | 0.0042 | -0.0069 | 0.0041 |
|  | Lingual_R | -0.0025 | 0.0170 |  |  | -0.0022 | 0.0497 | -0.0070 | 0.0021 |
|  | Occipital_Sup_L |  |  |  |  | -0.0038 | 0.0014 | -0.0080 | 0.0018 |
|  | Occipital_Sup_R | -0.0025 | 0.0286 |  |  |  |  | -0.0060 | 0.0039 |
|  | Occipital_Mid_L | -0.0025 | 0.0266 |  |  |  |  | -0.0068 | 0.0012 |
|  | Fusiform_L | -0.0026 | 0.0222 |  |  | -0.0022 | 0.0193 | -0.0064 | 0.0014 |
|  | Fusiform_R | -0.0030 | 0.0038 |  |  |  |  | -0.0064 | 0.0003 |
|  | Postcentral_L |  |  |  |  |  |  | -0.0028 | 0.0996 |
|  | Postcentral_R |  |  |  |  |  |  | -0.0044 | 0.0090 |
|  | Parietal_Sup_L |  |  |  |  |  |  | -0.0051 | 0.0027 |
|  | Parietal_Sup_R |  |  |  |  |  |  | -0.0045 | 0.0026 |
|  | Parietal_Inf_L |  |  |  |  | -0.0014 | 0.0139 | -0.0037 | 0.0027 |
|  | Parietal_Inf_R |  |  |  |  |  |  | -0.0040 | 0.0082 |
|  | Angular_L |  |  |  |  | -0.0017 | 0.0389 | -0.0037 | 0.0342 |
|  | Precuneus_L | -0.0022 | 0.0372 |  |  | -0.0021 | 0.0125 | -0.0067 | 0.0001 |
|  | Precuneus_R | -0.0024 | 0.0222 |  |  | -0.0026 | 0.0067 | -0.0073 | 0.0003 |
|  | Paracentral_Lobule_L |  |  |  |  |  |  | -0.0051 | 0.0251 |
|  | Paracentral_Lobule_R | -0.0048 | 0.0126 |  |  | -0.0033 | 0.0140 | -0.0070 | 0.0163 |
|  | Caudate_L | -0.0021 | 0.0414 |  |  |  |  | -0.0044 | 0.0201 |
|  | Temporal_Sup_L | -0.0029 | 0.0020 |  |  |  |  |  |  |
|  | Temporal_Sup_R |  |  |  |  |  |  | -0.0045 | 0.0102 |
|  | Temporal_Pole_Sup_R | -0.0023 | 0.0304 | -0.0068 | 0.0062 |  |  | -0.0062 | 0.0024 |
|  | Temporal_Mid_L |  |  |  |  | -0.0015 | 0.0490 | -0.0033 | 0.0490 |
|  | Temporal_Mid_R | -0.0019 | 0.0324 | -0.0048 | 0.0244 |  |  | -0.0044 | 0.0054 |
|  | Temporal_Pole_Mid_R | -0.0029 | 0.0072 | -0.0058 | 0.0285 |  |  | -0.0053 | 0.0069 |
|  | Temporal_Inf_L |  |  |  |  |  |  | -0.0036 | 0.0301 |
|  | Temporal_Inf_R | -0.0028 | 0.0038 | -0.0049 | 0.0381 |  |  | -0.0043 | 0.0106 |
| Regional efficiency | Precentral_L |  |  |  |  |  |  | -0.0028 | 0.0108 |
|  | Precentral_R | -0.0020 | 0.0128 |  |  | -0.0026 | 0.0010 | -0.0046 | 0.0079 |
|  | Frontal_Sup_Orb_R |  |  |  |  | -0.0021 | 0.0128 | -0.0044 | 0.0163 |
|  | Frontal_Mid_L |  |  | -0.0056 | 0.0049 | -0.0018 | 0.0105 |  |  |
|  | Frontal_Mid_R |  |  | -0.0045 | 0.0321 | -0.0018 | 0.0384 | -0.0044 | 0.0180 |
|  | Frontal_Inf_Oper_L |  |  |  |  |  |  | -0.0027 | 0.0291 |
|  | Frontal_Inf_Tri_R |  |  | -0.0059 | 0.0134 |  |  | -0.0043 | 0.0110 |
|  | Supp_Motor_Area_L |  |  | -0.0045 | 0.0272 | -0.0017 | 0.0026 | -0.0029 | 0.0232 |
|  | Supp_Motor_Area_R |  |  |  |  | -0.0018 | 0.0031 | -0.0040 | 0.0015 |
|  | Frontal_Sup_Medial_L | -0.0026 | 0.0062 | -0.0067 | 0.0024 | -0.0025 | 0.0025 | -0.0048 | 0.0062 |
|  | Rectus_L |  |  | -0.0047 | 0.0386 |  |  | -0.0040 | 0.0174 |
|  | Rectus_R |  |  | -0.0055 | 0.0091 |  |  |  |  |
|  | Cingulum_Ant_L | -0.0023 | 0.0047 |  |  | -0.0017 | 0.0090 | -0.0033 | 0.0214 |
|  | Cingulum_Ant_R |  |  |  |  | -0.0028 | 0.0001 | -0.0040 | 0.0117 |
|  | Cingulum_Mid_L | -0.0019 | 0.0124 |  |  | -0.0014 | 0.0180 | -0.0026 | 0.0453 |
|  | Cingulum_Post_L |  |  |  |  | -0.0023 | 0.0014 | -0.0041 | 0.0085 |
|  | Hippocampus_L |  |  | -0.0051 | 0.0273 |  |  | -0.0048 | 0.0062 |
|  | Hippocampus_R |  |  | -0.0057 | 0.0176 |  |  | -0.0047 | 0.0114 |
|  | ParaHippocampal_L |  |  |  |  |  |  | -0.0043 | 0.0055 |
|  | ParaHippocampal_R |  |  |  |  |  |  | -0.0048 | 0.0059 |
|  | Calcarine_L | -0.0028 | 0.0034 |  |  | -0.0020 | 0.0271 | -0.0057 | 0.0028 |
|  | Calcarine_R |  |  |  |  | -0.0026 | 0.0065 | -0.0070 | 0.0005 |
|  | Cuneus_L | -0.0024 | 0.0048 |  |  | -0.0017 | 0.0491 | -0.0044 | 0.0131 |
|  | Cuneus_R | -0.0020 | 0.0115 |  |  | -0.0018 | 0.0330 | -0.0056 | 0.0017 |
|  | Lingual_L | -0.0024 | 0.0102 |  |  | -0.0023 | 0.0161 | -0.0060 | 0.0038 |
|  | Lingual_R | -0.0028 | 0.0055 |  |  | -0.0029 | 0.0030 | -0.0052 | 0.0145 |
|  | Occipital_Sup_L |  |  |  |  | -0.0023 | 0.0055 | -0.0068 | 0.0001 |
|  | Occipital_Sup_R | -0.0022 | 0.0120 |  |  |  |  | -0.0064 | 0.0001 |
|  | Occipital_Mid_L | -0.0025 | 0.0367 |  |  | -0.0022 | 0.0123 | -0.0066 | 0.0003 |
|  | Fusiform_L |  |  |  |  | -0.0018 | 0.0360 | -0.0048 | 0.0074 |
|  | Parietal_Sup_L |  |  |  |  |  |  | -0.0046 | 0.0346 |
|  | Parietal_Sup_R | -0.0031 | 0.0013 |  |  | -0.0021 | 0.0324 | -0.0056 | 0.0070 |
|  | Parietal_Inf_L |  |  |  |  | -0.0011 | 0.0300 | -0.0036 | 0.0007 |
|  | SupraMarginal_R |  |  |  |  |  |  | -0.0020 | 0.0358 |
|  | Angular_L |  |  |  |  |  |  | -0.0025 | 0.0070 |
|  | Precuneus_L | -0.0024 | 0.0076 |  |  | -0.0019 | 0.0366 | -0.0063 | 0.0009 |
|  | Precuneus_R | -0.0023 | 0.0061 |  |  | -0.0019 | 0.0225 | -0.0060 | 0.0007 |
|  | Paracentral_Lobule_L | -0.0034 | 0.0000 |  |  | -0.0023 | 0.0013 | -0.0051 | 0.0007 |
|  | Paracentral_Lobule_R | -0.0025 | 0.0022 |  |  | -0.0015 | 0.0144 | -0.0048 | 0.0001 |
|  | Caudate_L | -0.0026 | 0.0024 |  |  | -0.0017 | 0.0257 | -0.0053 | 0.0006 |
|  | Thalamus_R | -0.0019 | 0.0084 | -0.0036 | 0.0360 | -0.0018 | 0.0168 | -0.0052 | 0.0009 |
|  | Heschl_L |  |  |  |  | -0.0013 | 0.0467 |  |  |
|  | Temporal_Pole_Sup_R | -0.0016 | 0.0378 | -0.0040 | 0.0299 |  |  | -0.0042 | 0.0087 |
|  | Temporal_Mid_R | -0.0017 | 0.0351 |  |  |  |  | -0.0040 | 0.0084 |
|  | Temporal_Pole_Mid_R |  |  | -0.0056 | 0.0063 |  |  | -0.0039 | 0.0197 |
|  | Temporal_Inf_L |  |  |  |  |  |  | -0.0044 | 0.0210 |

MS, multiple sclerosis; NMOSD, neuromyelitis optica spectrum disorders; EDSS, Expanded Disability Status Scale

We showed only significant results (*p* < 0.05).

**Table S4** Disrupted edges associated with clinical parameters (disease duration and EDSS) in the MS and NMOSD groups

| Subnetwork | Edges | MS | | | | | | NMOSD | | | | | |
| --- | --- | --- | --- | --- | --- | --- | --- | --- | --- | --- | --- | --- | --- |
|  |  | Disease duration | | | EDSS | | | Disease duration | | | EDSS | | |
|  |  | beta | *p* value | | beta | *p* value | | beta | *p* value | | beta | *p* value | |
| 1 | Hippocampus_L-ParaHippocampal_L | -0.0042 | | 0.0068 |  | |  | N/A | | N/A | N/A | | N/A |
|  | Hippocampus_L-Fusiform_L | -0.0060 | | 0.0349 |  | |  | N/A | | N/A | N/A | | N/A |
|  | Lingual_L-Temporal_Inf_L |  | |  |  | |  |  | |  | -0.0121 | | 0.0112 |
|  | Angular_L-Temporal_Mid_L |  | |  |  | |  | -0.0044 | | 0.0044 |  | |  |
|  | Temporal_Mid_L-Temporal_Inf_L |  | |  | -0.0059 | | 0.0479 |  | |  |  | |  |
|  | Parietal_Sup_L-Precuneus_R | -0.0196 | | 0.0111 |  | |  | N/A | | N/A | N/A | | N/A |
|  | Precuneus_L-Precuneus_R | -0.0044 | | 0.0315 |  | |  | -0.0062 | | 0.0005 | -0.0128 | | 0.0008 |
|  | Calcarine_R- Occipital_Sup_L |  | |  |  | |  | -0.0178 | | 0.0023 |  | |  |
|  | Cuneus_L-Occipital_Sup_L | -0.0048 | | 0.0052 |  | |  | N/A | | N/A | N/A | | N/A |
| 2 | Hippocampus_R-ParaHippocampal_R | -0.0038 | | 0.0131 |  | |  | N/A | | N/A | N/A | | N/A |
|  | Hippocampus_R-Fusiform_R | -0.0040 | | 0.0430 |  | |  | N/A | | N/A | N/A | | N/A |
|  | Hippocampus_R-Thalamus_R | -0.0058 | | 0.0041 |  | |  | N/A | | N/A | N/A | | N/A |
|  | ParaHippocampal_R-Fusiform_R | -0.0044 | | 0.0096 |  | |  | N/A | | N/A | N/A | | N/A |

MS, multiple sclerosis; NMOSD, neuromyelitis optica spectrum disorders; EDSS, Expanded Disability Status Scale; N/A, not applicable

We showed only significant results.

**Supplementary Figure S1** White-matter lesion probability map (A) for the multiple sclerosis (MS) group (*N*=66) and (B) for the neuromyelitis optica spectrum disorder (NMOSD) group (*N*=50). (C) Map indicating differences between the MS and NMOSD maps. The color scale indicates the probability range for each lesion. Montreal Neurological Institute standard space *x*, *y*, and *z* coordinates are displayed in the right upper corner of each subplot. In panel C, red indicates where lesions are more likely in MS than NMOSD, blue indicates where lesions are more likely in NMOSD than MS, and the black vertical line indicates no difference between groups.

**Supplementary Figure S2** Bar graphs for connection edge weights within subnetworks identified through network-based statistics. Each bar represents the fractional anisotropy (FA) adjusted for age and sex effects. Data are mean and 95% confidence intervals. HC, healthy controls.

ParaHippocampal, Parahippocampal gyrus; Calcarine, Calcarine fissure; Lingual, Lingual gyrus ; Occipitral_Sup, Superior occipital gyrus; Occipital_Mid, Middle occipital gyrus; Fusiform, Fusiform gyrus; Parietal_Sup, Superior parietal gyrus; Angular, Angular gyrus; Temporal_Sup, Superior temporal gyrus; Temporal_Pole_Sup, Temporal pole: superior temporal gyrus; Temporal_Mid, Middle temporal gyrus; Temporal_Pole_Mid, Temporal pole: middle temporal gyrus; Temporal_Inf, Inferior temporal gyrus; R, right; L, left;
