# Supplementary figures and images for: Disrupted structural network of inferomedial temporal regions in relapsing–remitting multiple sclerosis compared with neuromyelitis optica spectrum disorder
Source: Sci Rep. 2022 Mar 25;12:5152. doi: 10.1038/s41598-022-09065-4 (PMC8956623; doi:10.1038/s41598-022-09065-4)

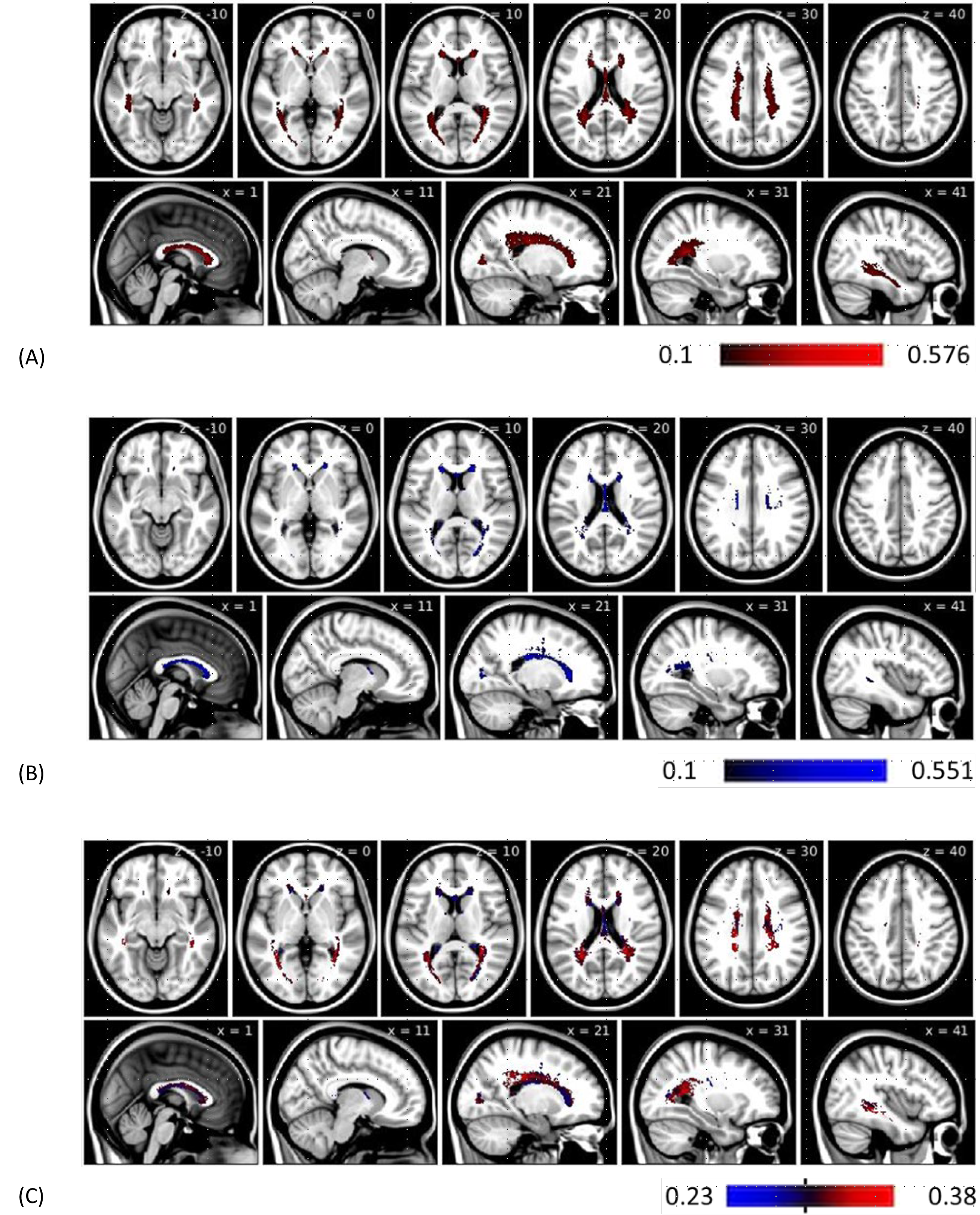

Supplement: Supplementary file 2 — Supplementary Figure S1. [file 41598_2022_9065_MOESM2_ESM.tif]
